# Prospective analysis of STRATAFIX™ symmetric PDS plus suture for fascial closure in spinal surgery: a pilot study

**DOI:** 10.1007/s10143-024-02671-y

**Published:** 2024-08-17

**Authors:** Steven R. Glener, Pious D. Patel, Stephanie N. Serva, Dwight Mitchell Self, Joshua E. Heller

**Affiliations:** Department of Neurological Surgery, Thomas Jefferson University Hospital, Philadelphia, Pennsylvania USA

**Keywords:** Wound closure, Fascia, Spine surgery, Wound healing, Suture

## Abstract

Wound closure is an integral part of every spinal procedure. Effective and secure wound closure is paramount in the prevention of infection, wound dehiscence and the preservation of cosmesis. Barbed suture technologies such as STRATAFIX™ Symmetric have been studied and are used in a variety of specialties, including obstetrics and orthopedic surgery, but is underutilized in neurosurgery. This study aims to assess the time and rate of closure using STRATAFIX™ Symmetric technology for fascial closure and compare this method to the more traditionally used method of fascial closure using braided absorbable sutures below the epidermis. 20 patients were recruited for the study. 10 patients underwent fascial approximation with braided absorbable sutures and definitive fascial closure with STRATAFIX™ Symmetric. In the control group, fascial closure was completed entirely with interrupted braided absorbable stitches. Patients assigned to STRATAFIX™ Symmetric group had shorter mean time for fascial closure, faster rate of average fascial closure, and lower number of total sutures used. The use of barbed suture technology such as STRATAFIX™ Symmetric may reduce the time to closure in thoracolumbar spine surgery without increasing the risk of adverse events. This pilot study forms the framework for a larger randomized, controlled trial appropriately powered for such an analysis.

## Introduction

Wound closure is an integral part of every spinal procedure. Effective and secure wound closure is paramount in the prevention of infection, wound dehiscence and the preservation of cosmesis. While there is some variation in specific wound closure method, many spine surgeons choose to close wounds in a methodical, layered fashion [1]. Various sutures are utilized in each layer. Conventionally, braided absorbable suture is used at or below the dermis and a litany of other closure techniques may be used at the epidermis, i.e., monofilament absorbable suture, nylon suture, staples. The process of closing a wound can be tedious and time-consuming, which can elicit sub-optimal closure and create opportunity cost from time spent in operating room [2, 3]. The literature has shown that the use of barbed suture technology is associated with less suturing time, faster wound closure, lower hospital costs, and fewer postoperative complications when compared to the use of traditional sutures [2–10].

Barbed suture technologies such as STRATAFIX™ Symmetric have been studied and are used in a variety of specialties, including obstetrics and orthopedic surgery [7, 11], but is underutilized in neurosurgery. STRATAFIX™ Symmetric uses barbs to secure the suture to the tissue in the absence of fixating knots [4]. The proposed benefit of the suture is that it evenly distributes tension along the length of an incision, with the added benefit of providing an antimicrobial coating in the STRATAFIX™ Symmetric variety of suture.

The primary aim of this study is to assess the time and rate of closure using the STRATAFIX™ Symetric technology for fascial closure and compare this method to the more traditionally used method of fascial closure using braided absorbable sutures below the epidermis. Decreased time to closure directly translates into decreased operating room time, and thus increased savings, especially for high-volume institutions. The secondary aim of this study is to determine the risk for surgical complications using STRATAFIX™ Symmetric within thoracolumbar spine surgery specifically. Previous studies in other specialties have shown no significant difference in rate of adverse consequences when utilizing barbed sutures [3, 4, 6, 8, 12]. To assess this aim, patients will be evaluated for wound dehiscence, SSI, deep infection, and 30-day rate of readmission for wound infection.

## Design & methods

### Patient selection

A total of 20 patients were recruited for the study. Pre-operatively, the patients were randomly assigned to either the conventional fascial closure method with braided absorbable sutures, or fascial closure using the STRATAFIX™ Symmetric barbed suture. Our research team screened for patients that were [1] over the age of 18 years old; [2] undergoing posterior thoracolumbar decompression, with or without fusion, of at least 3 vertebral levels; [3] anticipated skin incision of at least 15 cm; [4] able to sign informed consent with ability to follow research protocol. Exclusion criteria included [1] patients with current spinal infection [osteomyelitis]; [2] patient with co-morbid bacteremia; [3] patients with poorly controlled diabetes; [4] patients with severe malnourishment; [5] incidental durotomy during surgery. Patient demographics including age, gender, and comorbid conditions including hypertension, hyperlipidemia, and BMI (obesity > 30 kg/m^2^) were also documented (Table 1). While designed as a pilot study, a power calculation was additionally performed by a standard power analysis with 80% power to detect a minimum difference of 0.5 cm/min (presumed control group speed of 2 cm/min with standard deviation of 0.4, as suggested by retrospective institutional review of historical cases) with a *p*-value of 0.05.

**Table 1:** Patient demographic data

Patient Demographics		STRATAFIX™ Symmetric [*n* = 10]	Braided absorbable [*n* = 10]	*P*-value
Age, years (mean (SD))		$66.2 \left(8.4\right)$	62.8 (12.8)	0.491
Sex (n (%))	Male	4 (40%)	4 (40%)	1.000
	Female	6 (60%)	6 (60%)	
BMI (mean (SD))		28.5 (6.8)	32.0 (5.7)	0.228
Hypertension		10 (100%)	9 (90%)	0.331
Hyperlipidemia		7 (70%)	7 (70%)	1.000

### Procedures

Both the patients and data analysts were blinded to the type of suture utilized during the surgery. All patients underwent general anesthesia, and the dissection was completed in the same manner for all patients. Surgery was completed by two surgeons operating simultaneously. The surgeries included in this study were done by the same cohort of qualified surgeons at one institution, which included both resident and attending neurosurgeons.

**Fig. 1:**
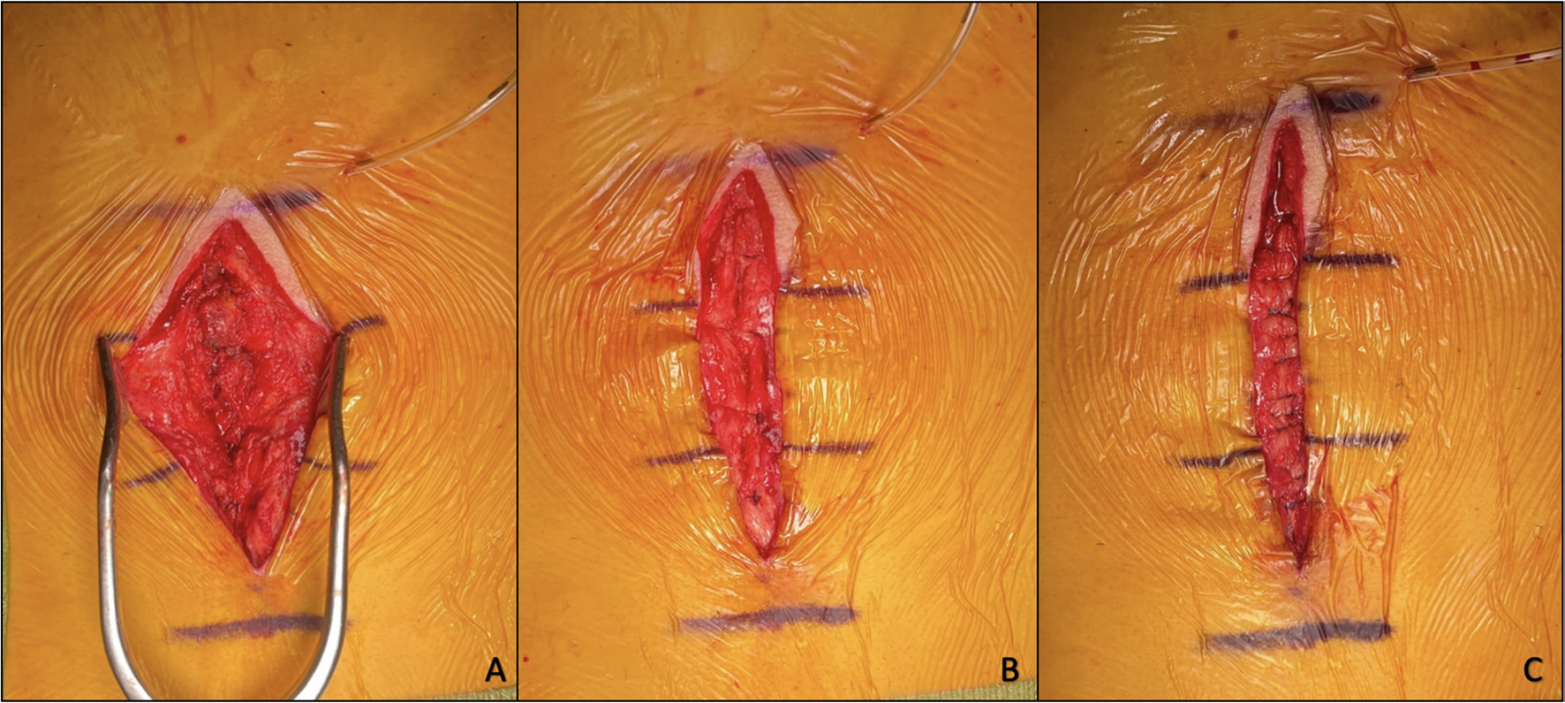
**A** Muscle has been approximated with braided absorbable suture. A retractor is used to better delineate the fascial layer so that it can be adequately closed. **B** Fascia has been approximated using three (3) braided absordable suture in preparation for use of barbed STRATAFIX for definitive closure of the fascial layer. **C** Complete fascial closure with 3 braided absordable sutures, in addition to barbed STRATAFIX

After completion of the spinal procedure, the lead surgeon verbalized to operating room staff when he or she is beginning closure of the fascia. The time from which the first stitch is started through completion of the final stitch is documented. Wound closures were done systematically, beginning with muscle approximation with braided absorbable stitches, followed by fascial approximation with braided absorbable sutures and definitive fascial closure with STRATAFIX™ Symmetric. When performing closure with STRATAFIX™ Symmetric, the surgeons utilized a standardized closure technique in which the suture length-to-wound length (SL/WL) ratio was 4:1, which was acquired with small stitches pulled tightly. Specifically, the STRATAFIX™ suture that was used was the 18-inch Symmetric PDS Plus, knotless tissue control device with antimicrobial coating, size 1–0 on a tapered CTX needle made by Ethicon. In the control group, fascial closure was completed entirely with interrupted braided absorbable stitches, specifically 18-inch, size 0 Vicryl suture on an MO-4 needle made by Ethicon. In the experimental group, fascial closure was done via fascial approximation with 2–3 interrupted braided absorbable sutures followed by barbed STRATAFIX™ Symmetric (Fig. 1). The approximating sutures were placed in line with the superficial marks made on the skin, in order to ensure an aesthetic closure of each layer, preventing the need for tissue mobilization at more superficial layers of closure. In both groups, braided absorbable 2–0 Vicryl sutures on a CP-2 needle were used to close the deep dermal layer, and staples were used for epidermis closure. Additional steps that occurred after the skin was closed, including stitching the drain in place, injection of local anesthetic, and dressing the wound were not accounted for.

Patient’s surgical site was monitored and assessed for infection and cerebrospinal fluid [CSF] leakage at the time of surgery, and at post-operative discharge, 2 weeks, 6 weeks, 3 months and 6 months. A physical exam and assessment of adverse events was also performed at these time points.

## Results

The average age of patients assigned to the STRATAFIX™ Symmetric group was $66.2 (\text{SD }8.4)$ years, and 62.8 (SD 12.8) years for the conventional suture group (*P* = 0.491). Among enrolled patients, 60% of which identified as female (*n* = 12), 40% identified as male (*n* = 8), with 6 females and 4 males in each group. The mean BMI amongst participants in the STRATAFIX™ Symmetric group was 28.5 (SD 6.8) [kg/m^2^], in comparison to the conventional suture group in which the average BMI was 32.0 (SD 5.7) (kg/m^2^) (*P* = 0.228). Five participants in each group had BMIs within obese/morbid obesity range. In addition to obesity, the most common comorbidities of the patient population were hypertension, and hyperlipidemia. All patients in the STRATAFIX™ Synmetric group and 9 of 10 patients in the braided absorbable group had a history of hypertension. In addition, 7 of 10 patients in each of the two groups had co-morbid hyperlipidemia.

The average length of incision in the STRATAFIX™ Symmetric group was 27.0 (SD 9.2) cm compared to 30.5 (SD 8.7)cm in the braided absorbable group (*P* = 0.401). The mean time for fascial closure using STRATAFIX™ Symmetic was 11.1 (SD 4.4) minutes, compared to the mean time for fascial closure in the braided absorbable group of 15.1 (SD 6.6) minutes (*P* = 0.128). Discrepancy in incision length between the two groups was accounted for by calculating the rate of closure in cm per minute. The average rate of fascial closure in the STRATAFIX™ Symmetric group was 2.7 (SD 0.9)cm per minute. Comparatively, the average rate of fascial closure in the braided absorbable group was 2.2 (SD 0.8)cm per minute (*P* = 0.286). Of note, the total time from start of closure from first muscular stitch to end of skin closure was 34.3 (SD 8.9)minutes in the fascial STRATAFIX™ Symmetric group, compared to 39.1 (SD 16.6)minutes in the braided absorbable group (*P* = 0.431). Notably, the average number of sutures used for fascial closure in the STRATAFIX™ Symmetric group was 6.1 sutures (includes Vicryl fascial approximation sutures), compared to 35.1 sutures in the facial closure with braided absorbable sutures (*P* < 0.001) (Table 2).

**Table 2:** Comparative outcomes for STRATAFIX™ symmetric group vs control group

	STRATAFIX™ [*n* = 10]	Braided absorbable [*n* = 10]	*P*-value
Fascial closure time, min (mean (SD))	11.1 (4.4)	15.1 (6.6)	0.128
Total closure time, min (mean (SD))	34.3 (8.9)	39.1 (16.6)	0.431
Mean incision length, cm (mean (SD))	27.0 (9.2)	30.5 (8.7)	0.401
Fascial closure rate, cm/min (mean (SD))	2.7 (0.9)	2.2 (0.8)	0.286
Number of sutures used, count (mean (SD))	6.1 (4.6)	35.1 (11.7)	< 0.001

At our institution, the cost for a single STRATAFIX™ Symmetric is $20.84. In the United States, the mean cost of operating room time has been reported to be $46.04 per minute [13]. Utilization of STRATAFIX™ Symmetric may result in overall cost savings of up to $220.99 per case, assuming a 4.8 min difference in fascial closure time.

Post-operatively, patients were monitored for wound dehiscence, surgical site infection, and 30-day rate of readmission for wound infection over the course of 6 months. Zero patients from either group experienced any of these adverse events after surgery.

## Discussion

This pilot study showed that the use of STRATAFIX™ Symmetric suture technology trends towards a reduced time to closure in comparison to the traditional method of closure with braided absorbable suture. In the STRATAFIX™ Symmetric group, the average time for fascial closure was 11.1 min, and the average rate of fascial closure was 2.7 cm/min, compared to the braided absorbable group, in which the average time for fascial closure was 15.1 min and the average rate of closure was 2.2 cm/min. However, this difference was not statistically significant, likely due to the small sample size of the study.

Importantly, there was a statistically significant difference in number of sutures required for fascial closure, 6.1 sutures versus 35.1 sutures [*P* < 0.001]. Reduced number of sutures facilitates faster post-surgical material counts by operating room staff. Additionally, reducing the number of sutures that are passed to and from the surgeon decreases the likelihood of accidental needle stick.

Decreasing closure time directly translates to decreased operating room time and increased cost savings for high-volume institutions [13–15]. At a large-scale center for thoracolumbar spine surgery, incremental improvements in closure time can generate significant cost savings.

Furthermore, the two groups were similar with respect to the rate of adverse events. None of the patients in either group experienced wound dehiscence, surgical site infection, or required readmission for wound infection within 30 days post-operatively.

Although this study suggests the safety and efficacy of STRATAFIX™ Symmetric in thoracolumbar spine surgery, it has limitations. The sample size was small, and the study only evaluated short-term outcomes over a period of six months. Future studies with larger sample sizes and longer follow-up periods would be needed to confirm the safety and efficacy of STRATAFIX™ Symmetric suture technology in.

## Limitations

This study is limited by its small sample size, which inherently reduces the power of the study and increases the margin of error. Further prospective studies with larger patient populations are needed to validate these findings.

## Conclusion

In conclusion, the use of barbed suture technology such as STRATAFIX™ Symmetric may reduce the time to closure in thoracolumbar spine surgery without increasing the risk of adverse events. This pilot study forms the framework for a larger randomized, controlled trial appropriately powered for such an analysis. The implications from reduced time to closure may be particularly beneficial for high-volume institutions where time in the operating room directly impacts cost savings.

## Data Availability

No datasets were generated or analysed during the current study.
